# Exploring non-rapid eye movement sleep substages in rats to develop biomarkers for depression

**DOI:** 10.1093/sleep/zsad117

**Published:** 2023-04-21

**Authors:** Alyssa Wiest, Shinjae Chung

**Affiliations:** Department of Neuroscience, Chronobiology, and Sleep Institute, Perelman School of Medicine, University of Pennsylvania, Philadelphia, PA, USA; Department of Neuroscience, Chronobiology, and Sleep Institute, Perelman School of Medicine, University of Pennsylvania, Philadelphia, PA, USA

The bidirectional relationship between sleep and stress is well established. Both animal models and human studies have demonstrated that stress can disturb the sleep architecture in many ways, leading to rapid eye movement (REM) sleep abnormalities and fragmented non-REM (NREM) sleep [1–6]. A majority of patients with depression suffer from sleep disturbances, with approximately 80% of individuals suffering from insomnia [7]. When left untreated, sleep disturbances increase the risk of developing psychiatric disorders and, in fact, often precede their clinical onset [8–12]. While disturbed sleep was once considered a secondary symptom of post-traumatic stress disorder (PTSD), a growing body of research indicates that sleep disturbances are not only prevalent in individuals with PTSD but are also a core feature of the disorder [13]. These sleep disturbances include difficulty initiating and maintaining sleep and recurring nightmares [14, 15]. However, not all individuals exposed to trauma develop PTSD, raising questions about the underlying factors that contribute to resilience [16]. While changes in the sleep architecture following stress may result from interactions between stress and sleep regulatory circuits, the underlying mechanisms are not yet fully understood. Elucidating the sleep biomarkers in vulnerable populations will facilitate the identification of the underlying neural mechanisms and provide novel therapeutic interventions prior to the onset of psychiatric disorders like depression and PTSD.

In the current issue of *SLEEP* (2023;46(7):zsad068. doi: 10.1093/sleep/zsad068), Claverie et al. introduced a novel method to subclassify NREM sleep in rats into three stages (N-S1, N-S2, and N-S3), allowing for a more direct comparison with human sleep stages. While NREM sleep is typically viewed as a homogeneous state in rodents, it encompasses multiple electrophysiological features including delta and theta waves, sleep spindles, hippocampal ripples, slow oscillations, and infraslow rhythms [17]. This suggests that NREM sleep in rodents could be further classified into distinct substages which likely have different neural correlates and are potentially involved in various functions of sleep. Human sleep is largely divided into NREM and REM sleep, with NREM sleep being further divided into substages N1, N2, and N3 [18]. In humans, N1 is the lightest stage of sleep and is distinguished by the presence of low-amplitude, mixed-frequency activity. In Claverie et al., the N-S1 stage is similarly the lightest sleep stage in rats as it has the lowest relative delta power and proportion of sleep spindles when compared to the other NREM sleep stages. Human stage 2 sleep constitutes about 45%–55% of total sleep and is characterized by the presence of K-complexes and sleep spindles. Consistent with human N2 sleep, Claverie et al. found that rat N-S2 had the highest proportion of sleep spindles compared to all three stages. Human N3, also called slow-wave sleep, is the deepest NREM sleep stage. It comprises 25% of total sleep and is dominated by low-frequency, high-amplitude delta waves. The authors observed that the relative delta power is highest during rat N-S3 sleep. The progressive deepening of N-S1 to N-S3 sleep provides further support that these stages share similarities with human NREM sleep stages, although they may not be completely homologous. A few studies have also endeavored to categorize rodent sleep into distinct stages. Lacroix et al. identified three stages of NREM sleep in mice [19]. Another study in rats subclassified NREM sleep into three stages: NREM1, NREM2, and transition sleep [20]. The latter stage is defined by a prominent theta rhythm mixed with high-amplitude spindles that occur before transitioning to REM sleep [20–22].

Based on their subclassification of NREM sleep, Claverie et al. were able to identify distinct sleep biomarkers in two groups of rats classified as either vulnerable or non-vulnerable to depression based on their serum brain-derived neurotrophic factor levels 30 days following the last day of social defeat stress [23]. The validity of using serum brain-derived neurotrophic factor levels as an indicator of vulnerability was supported by earlier research that employed various measures of depression-like behavior, including the sucrose preference test, forced swim test, and hypothalamic-pituitary-adrenal axis activity [24, 25].

In humans, depression is associated with a prolonged latency to falling asleep, fragmented NREM sleep due to an increase in microarousals, and a decrease in N2 and N3 sleep [7]. Furthermore, one of the prominent features observed in electroencephalogram recordings is a reduced latency to REM sleep and an elevated REM density [26]. These abnormalities are present during depressive episodes and persist in remission, suggesting they might be useful as vulnerability biomarkers. Therefore, identifying baseline sleep biomarkers in the electroencephalogram could provide an early indication of vulnerability and aid in the prevention and management of depression. Analyzing the baseline recordings (referred to as early vulnerability), the authors noted that vulnerable rats had a longer N-S1 duration and shorter N-S3 duration when compared to non-vulnerable rats. The authors found that, during recovery sleep after social defeat stress (referred to as late vulnerability), vulnerable rats had longer and more frequent N-S1 episodes, accompanying a higher number of transitions from N-S1 to wake as well as an increase in microarousals. These results imply that vulnerable rats have more fragmented NREM sleep, much like the sleep disturbances seen in humans with depression. Interestingly, the authors did not find significant REM sleep differences between vulnerable and non-vulnerable rats during their baseline or recovery sleep. Restraint stress has been shown to increase REM sleep in rodents, demonstrating that different types of stressors may disturb different aspects of sleep [27, 28]. Taken together, Claverie et al.’s work is a crucial foundation for guiding future biomarker studies and reexamining existing longitudinal data sets for other potential biomarkers.

In summary, Claverie et al.’s findings introduce a promising new method to subclassify NREM sleep in rats, which has the potential to facilitate more direct comparisons with human sleep stages and aid in the identification of early and late sleep biomarkers of vulnerability to depression. Given the highly variable impact of stress on sleep patterns between individuals, the identification of reliable sleep biomarkers for susceptibility to depression is crucial to allow for timely intervention. Previous studies have demonstrated that social stress impairs various aspects of sleep in rodents [29–32]. Investigating changes in the sleep microarchitecture, such as specific NREM sleep stages and impairments in various brain rhythms during sleep, can improve our understanding of how stress affects vulnerable populations during sleep. Expanding on these findings, identifying the neural circuit mechanisms that regulate specific features of sleep can offer novel therapeutic targets for improving sleep in vulnerable populations to potentially prevent the development of psychiatric disorders. A recent study in mice has shown that stress-induced sleep disturbances are partly mediated by the projections from noradrenergic neurons in the locus coeruleus to the preoptic area [33]. Improving sleep by activating preoptic neurons could increase resiliency to social stress, which highlights this circuit as a promising target for studying potential differences between vulnerable and non-vulnerable populations [34]. Identifying sleep circuits that are impacted by stress could pave the way for developing targeted interventions to prevent the onset of depression and other mood disorders in individuals who are most susceptible.
